# The Success of Cataract Surgery and the Preoperative Measurement of Retinal Function by Electrophysiological Techniques

**DOI:** 10.1155/2015/401281

**Published:** 2015-10-21

**Authors:** Jing An, Lei Zhang, Yusheng Wang, Zuoming Zhang

**Affiliations:** ^1^Department of Clinical Aerospace Medicine, Fourth Military Medical University, Xi'an 710032, China; ^2^Department of Ophthalmology, Xijing Hospital, Fourth Military Medical University, Xi'an 710032, China

## Abstract

*Purpose*. To study the effect of different electrophysiological methods to evaluate retinal function prior to cataract surgery. *Methods*. Cataract patients who had no significant other eye disease were chosen. VA, pattern visual evoked potential (PVEP), electroretinogram (ERG), and multifocal electroretinogram (mfERG) responses were measured from 150 cataract patients and 20 control subjects. *Results*. When the preoperative VA was more than 0.3 in cataract patients, the amplitude of PVEP was not significantly different between cataract and control subjects. The amplitude of central point mfERG was significantly lower in cataract patients compared with control group from HM to 0.8 of preoperative VA. The 95% confidence intervals (CIs) of the amplitudes of center point mfERG were calculated for a range of preoperative VA values. Most of the patients within 95% CI of the center point mfERG had a postoperative VA more than 0.5. *Conclusions*. The amplitude of central point mfERG in cataract patients was the most relevant parameter to the preoperative VA compared with PVEP and ERG. The 95% CI of the amplitude of central point mfERG for each level of VA could help to evaluate preoperative macular function which is used to predict the outcome of cataract surgery.

## 1. Introduction

Cataract is the major cause of blindness in the world. Cataract surgery and lens implantation is usually a successful treatment if the patient's retina is fully functional. However, 35 to 55% of patients had some visual functional impairment after cataract surgery (Snellen acuity of 0.67 or less) [1]. In 2011, the American Academy of Ophthalmology suggested that the decision to perform cataract surgery should be made based on the measurement of visual acuity (VA) as well as other functional measurements [2]. Preoperative testing such as intraocular pressure, slit lamp examination, color vision examination, B ultrasound examination, and visual field cannot evaluate retinal function comprehensively and routine physical examination before cataract surgery does not increase the safety of the surgery [3]. Cataract surgery improved the VA in the majority of the patients; however, it would be expected that the VA would not be improved in patients who have poor retinal and macular function prior to surgery. Preoperative measurement of retinal function is needed to provide information beyond that which could be obtained by routine ophthalmological examination.

Electrophysiology technologies have been used to examine retinal function prior to cataract surgery. Visual electrophysiology techniques such as pattern visual evoked potential (PVEP) and flash electroretinogram (ERG) are used in appraising the necessity of cataract surgery and postoperative visual function [4, 5]. PVEP is used to measure the functional integrity of the visual pathways from retina via the optic nerves to the visual cortex. Any abnormality of the optic pathways will affect the PVEP response. Therefore PVEP response is sensitive to lenticular opacity, but it misdiagnosed macular degeneration when patients suffer from cataract [6]. Moreover, individual variations are prevalent with light flash VEP. VA is associated with light focused on the fovea which encompasses approximately 5% of the central retinal area. The full field ERG measures whole retinal function. However, with cataract, central vision is impaired and full field ERG is not sensitive to the small change in the response of the fovea. Fovea function can be evaluated by multifocal electroretinogram (mfERG). mfERG is no longer used as a routine test for cataract patients. There are few studies that correlate PVEP, mfERG, and ERG with preoperative and postoperative VA. Also, there are no publications that relate mfERG and the degree of VA of cataract patients.

Our goal of this study was to examine the response of visual electrophysiology techniques and to compare the correlations with different degree of cataract by VA. In addition, the 95% CIs of the amplitudes of center point mfERG for a range of preoperative VA values were obtained that could be used to evaluate the outcome of surgery. And diagnostic values of ERG and mfERG in patients with cataract are valuable for evaluation of surgical outcome.

## 2. Methods

### 2.1. Subjects

A total of 150 subjects (277 cataractous eyes) aged 40–86 years with average age of 65.73 ± 10.43 years were used for this study. Of the cataractous eyes 150 were from women and 127 from men and 171 eyes received cataract surgery. There were no restrictions on the degree of cataract. The VA of all subjects was in the range −3.0 D to + 3.0 D and the subjects did not have other ophthalmic or general diseases other than cataract. As controls, we tested 20 eyes from participants that were recruited from the Xi Jing Hospital of Fourth Military Medicine University that did not have any pathology or ocular medical history and had a standard logarithmic VA better than 1.0. Their age ranged from 46 to 72 years and averaged 60.50 ± 7.76 years. All subjects received an eye examination including refraction, slit lamp, and indirect ophthalmoscope before and 2 days after cataract surgery. Cataract surgeries were performed using a clear corneal incision, phacoemulsification, and posterior chamber intraocular lens implantation. A foldable hydrophobic acrylic intraocular lens was implanted. The experimental procedures were approved by the Medical Ethics Committee of the Fourth Military Medicine University, Xi'an, China. The institute's ethics committee approved the study before the patients' records were analyzed. Patients and control subjects voluntarily joined this study with informed consent.

The data from the evaluation of 104 normal eyes from our ERGs database was studied in order to build normal ERG values by age. All cataract subjects (277 eyes) were chosen by the 80% amplitude of b-wave in scotopic 3.0 ERG response according to the normal data (Table 1) in different age groups.

### 2.2. Visual Acuity Test

A standard logarithmic VA chart was used to test the best corrected VA (GB11533-2011, Six-Six Vision Corp., China) two days before and after cataract surgery. The E characters were viewed at a distance of 5 meters and VA was based on the smallest line that could be read. If patients could not see any “E” characters at 5 m, VA was measured at 4 m, 3 m, 2 m, and 1 m and was calculated by the formula: VA = 0.1 *∗* distance/5. If patients could not see words at less than 1 m, the VA was measured if the patients could count fingers (CF); see hand movements (HM) or perceive light (LP) 30 centimeters away. Subjects were divided into eight groups according to their VA: HM, CF, 0.01 to 0.1, 0.1 to 0.2, 0.2 to 0.3, 0.3 to 0.5, 0.5 to 0.8, and more than 0.8 (Table 2).

### 2.3. PVEP

PVEP recordings were obtained using a commercial system (GOTEC-2011, GuoTe Biotechnical, Chongqing, China). The test method followed the ISCEV standards in 2009 update [7]. Pattern reversals were provided by black and white checks with reversal rates of 2.4 Hz. The field size was 15 deg, and the pattern element size was 30 min (1 cpd). The mean luminance of the stimulus was 180 cd/m^2^. The contrast was approximately 97%. The bandwidth was 1 to 75 Hz. The PVEP recordings were averaged by more than 64 sweeps. The electrode impedance was below 5 kΩ. In all measurements, the stimuli were presented monocularly. An Ag-AgCl electrode was placed in occipital lobe that was used as the active electrode. The reference and ground electrodes were attached to the forehead and earlobe, respectively.

### 2.4. ERG

The ERG test was conducted following the ISCEV standards in 2015 [8]. 1.0% tropicamide (Alcon, Fort Worth, USA) was placed into each eye. The ERG recording was not started until the pupils dilated to at least 7 mm in diameter. A jet electrode was used as the active electrode. Copper-cup electrodes were used for both the reference (located 1 cm from the outer canthus of the eye) and ground (located at the ear lobe) recording. Stimulations were produced using a full field stimulation globe with an LED light source positioned 15 cm away from the eye. A commercial system (GOTEC-2011, GuoTe Biotechnical, Chongqing, China) was used to measure dark-adapted 0.01 ERG, 3.0 ERG and 3.0 oscillatory potentials and light-adapted 3.0 ERG and 3.0 flicker. Strobe stimulus flashes were delivered in a Ganzfeld. We used a band pass of 1 Hz to 300 Hz except oscillatory potentials (OPs) (75–300 Hz). Stimulation luminance was set at 3.0 d·s·m^−2^ except the dark-adapted 0.01 ERG (0.01 d·s·m^−2^). Each stimulus condition was repeated two or three times, and waveforms were recorded for 500 ms. The electrode impedance was below 5 kΩ. The jet electrode was contacted cornea used as the active electrode. The Ag-AgCl reference and ground electrodes were attached to the outer canthus and earlobe, respectively.

### 2.5. mfERG

The mfERG test was conducted following the ISCEV standards in 2011 edition [9]. Pupils were dilated to at least 7 mm with 1.0% tropicamide (Alcon, Fort Worth, USA) placed in each eye. A jet electrode was used as the active electrode. Copper-cup electrodes were used for both the reference and ground recording. A GOTEC-2011 was used to run the mfERG software (GOTEC-2011, GuoTe Biotechnical, Chongqing, China) and the stimulus was presented on a Samsung liquid crystal diode (S22A300B). A scaled 61-hexagon stimulus pattern was selected and it provided approximately equal signal amplitude at each location. The viewing distance from the subject to the monitor was fixed at 25 cm. The optic angle was 27.7°. The degree of the center point mfERG (first ring) was 2.17°.

Stimuli were set at 105 cd·m^−2^ (white) and 1 cd·m^−2^ (black) with an average luminance of 53 cd·m^−2^ and the contrast was approximately 98%. The surrounding background light was dimmed. A band pass from 5 to 100 Hz and a gain of 100,000 were used. The stimulus frame rate was 60 Hz and the response signal was sampled at eight samples per frame with a sampling interval of 80 ms. The recording time for each stimulation cycle was approximately 42 s with intervals of about 5 s between segments. Any segments associated with blinks or eye movement were rejected and repeated immediately. The jet electrode was contacted cornea used as the active electrode. The Ag-AgCl reference and ground electrodes were attached to the outer canthus and earlobe, respectively.

### 2.6. Statistical Analysis

The amplitude and peak time of each waveform were measured by using a GOTEC-2011 (GuoTe Biotechnical, China). A one-way ANOVA followed by a least significant difference test was used for multiple-group comparisons. A Tamhane test was used if heterogeneity existed in the group. Pearson's coefficient was used to evaluate correlation. The data are presented as the mean ± the standard error of the mean. Figures were made using Origin 8.0 (OriginLab, Northampton, USA). Significance was accepted at the *P* < 0.05 level. All data were analyzed statistically using SPSS 16.0 software.

## 3. Results

### 3.1. ERG Response

150 patients (277 eyes) were chosen with different degrees of cataract, whose amplitude of b-wave in dark-adapted 3.0 ERG was 80% of the normal age matched control in our lab to ensure good retinal function (Table 1). The b-wave amplitude level chosen was more than 350 *μ*V, 330 *μ*V, 300 *μ*V, 280 *μ*V, and 260 *μ*V for the age ranges 40–50 years, 50–60 years, 60–70 years, 70–80 years, and more than 80 years, respectively.

**Figure 1:**
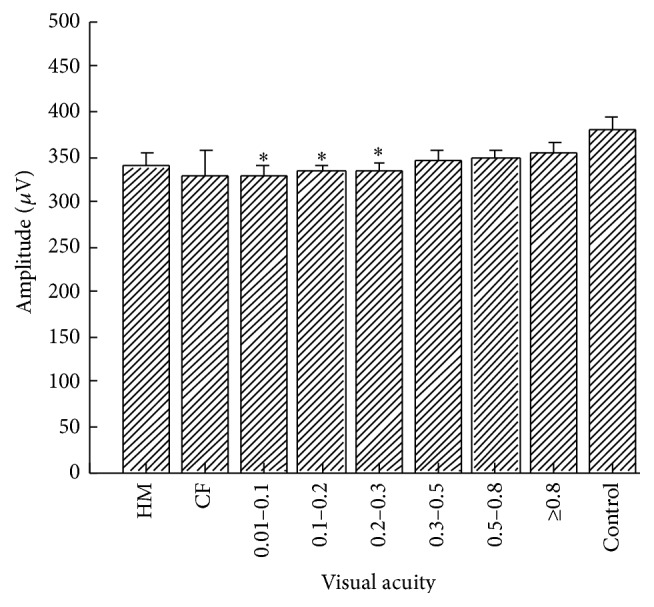
b-wave amplitudes of dark-adapted 3.0 ERG versus visual acuity for patients with cataract. ^*∗*^ *P* < 0.05 cataract patients versus the control group.

In all chosen cataract patients, the amplitude of the b-wave of the dark-adapted 3.0 ERG was not significantly different for each VA group. The three groups with cataracts had significantly lower b-wave amplitudes from 0.01 to 0.3 (*P*_0.01–0.1_ = 0.016; *P*_0.1–0.2_ = 0.013; *P*_0.2–0.3_ = 0.026, Figure 1) compared with the control group.

### 3.2. PVEP Response

**Figure 2:**
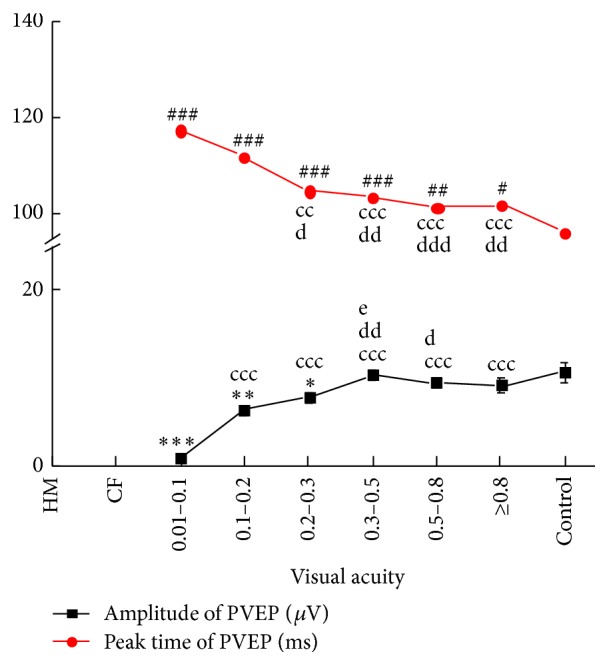
PVEP response versus the preoperative visual acuity of patients with cataracts. ^*∗∗∗*^ *P* < 0.001 comparison of the amplitude of PVEP between cataract patients and control subjects. ^#^ *P* < 0.05, ^##^ *P* < 0.01, and ^###^ *P* < 0.001 comparison of the peak time of PVEP between cataract patients and control subjects. ^cc^ *P* < 0.01, ^ccc^ *P* < 0.001 comparison between the 0.01–0.1 VA group and other cataract groups; ^d^ *P* < 0.05, ^dd^ *P* < 0.01, and ^ddd^ *P* < 0.001 comparison between the 0.1–0.2 VA group and other cataract groups; ^e^ *P* < 0.05 comparison between the 0.2–0.3 VA group and other cataract groups.

There was a significant delay (*P* < 0.05) of peak time of PVEP from patients with cataracts compared with the control group, but the amplitude of PVEP was significantly lower in only 0.01–0.3 VA groups compared with the control group (*P*_0.01–0.1_ = 0.000; *P*_0.1–0.2_ = 0.001; *P*_0.2–0.3_ = 0.014, Figure 2). When the VA was more than 0.3, differences were not significantly different compared with the control groups (*P*_0.3–0.5_ = 0.125; *P*_0.5–0.8_ = 0.351; *P*_0.8_ = 0.467, Figure 2).

The comparison of the responses in cataract groups showed the amplitude of PVEP was lower in 0.001–0.1 VA group compared with other VA groups (*P*_0.1–0.2_ = 0.000, *P*_0.2–0.3_ = 0.000, *P*_0.3–0.5_ = 0.000, *P*_0.5–0.8_ = 0.000, and *P*_0.8_ = 0.000, Figure 2). With the VA better than 0.2, the differences were not notable. The peak times of PVEP were delayed in the 0.01–0.1 and 0.1–0.2 VA groups compared with other VA groups (*P*_0.01–0.1  versus  0.2–0.3_ = 0.001, *P*_0.01–0.1  versus  0.3–0.5_ = 0.000, *P*_0.01–0.1  versus  0.5–0.8_ = 0.000, and *P*_0.01–0.1  versus  0.8_ = 0.000; *P*_0.1–0.2  versus  0.2–0.3_ = 0.024, *P*_0.1–0.2  versus  0.3–0.5_ = 0.002, *P*_0.1–0.2  versus  0.5–0.8_ = 0.000, and *P*_0.1–0.2  versus  0.8_ = 0.001, Figure 2). And, with the VA better than 0.2, there were no significant differences in these cataract groups.

### 3.3. mfERG Response

**Figure 3:**
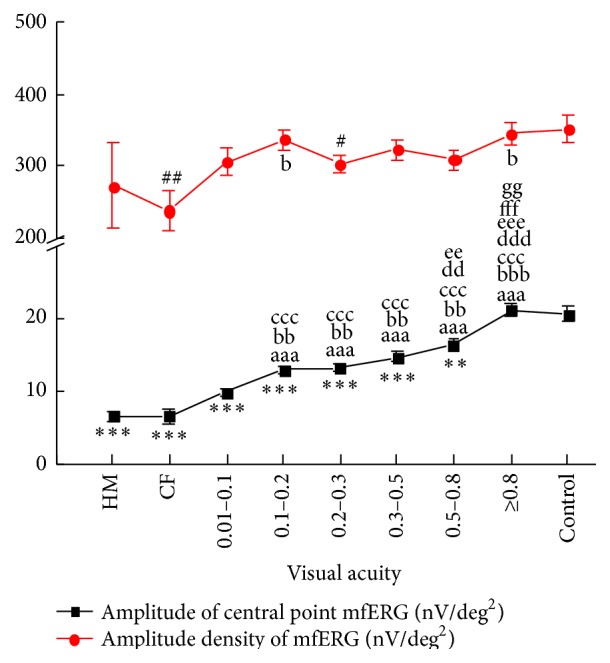
mfERG response of the cataract patients versus preoperative visual acuity. ^*∗∗*^ *P* < 0.01, ^*∗∗∗*^ *P* < 0.001 comparison of the amplitude of central point mfERG between cataract patients and control subjects. ^##^ *P* < 0.01, ^###^ *P* < 0.001 comparison of the amplitude density of mfERG between cataract patients and control subjects. ^aaa^ *P* < 0.001 comparison between the HM group and other cataract groups; ^b^ *P* < 0.05, ^bb^ *P* < 0.01, and ^bbb^ *P* < 0.001 comparison between the CF group and other cataract groups; ^cc^ *P* < 0.01, ^ccc^ *P* < 0.001 comparison between the 0.01–0.1 VA group and other cataract groups; ^dd^ *P* < 0.01, ^ddd^ *P* < 0.001 comparison between the 0.1–0.2 VA group and other cataract groups; ^ee^ *P* < 0.01, ^eee^ *P* < 0.001 comparison between the 0.2–0.3 VA group and other cataract groups; ^fff^ *P* < 0.001 comparison between the 0.3–0.5 VA group and other cataract groups; ^gg^ *P* < 0.01 comparison between the 0.5–0.8 VA group and other cataract groups.

The differences of amplitude density of mfERG were not significantly different between cataract patients grouped by VA and the control group, except for CF and 0.2–0.3 groups (*P*_CF_ = 0.005; *P*_0.2–0.3_ = 0.030, Figure 3). The amplitude of the central point mfERG was significantly lower in the HM to 0.8 VA groups compared with the control group (*P*_HM_ = 0.000; *P*_CF_ = 0.000; *P*_0.01–0.1_ = 0.000; *P*_0.1–0.2_ = 0.000; *P*_0.2–0.3_ = 0.000; *P*_0.3–0.5_ = 0.000; *P*_0.5–0.8_ = 0.004, Figure 3). When the VA was greater than 0.8, the amplitude of the central point mfERG in the cataract groups was similar to that of the control group (*P*_≧0.8_ = 0.674, Figure 3).

Comparing the responses of mfERG in different degree cataract groups, the amplitude density of mfERG in all groups, the differences were not notable. The amplitudes of central point mfERG keep low levels in the VA lower than 0.1 groups (*P*_HM  versus  0.1–0.2  _ = 0.000, *P*_HM  versus  0.2–0.3_ = 0.000, *P*_HM  versus  0.3–0.5_ = 0.000, *P*_HM  versus  0.5–0.8_ = 0.000, and *P*_HM  versus  0.8_ = 0.000; *P*_CF  versus  0.1–0.2_ = 0.033, *P*_CF  versus  0.2–0.3_ = 0.035, *P*_CF  versus  0.3–0.5_ = 0.005, *P*_CF  versus  0.5–0.8_ = 0.001, and *P*_CF  versus  0.8_ = 0.000; *P*_0.01–0.1  versus  0.1–0.2_ = 0.004, *P*_0.01–0.1  versus  0.2–0.3_ = 0.000, *P*_0.01–0.1  versus  0.3–0.5_ = 0.000, *P*_0.01–0.1  versus  0.5–0.8_ = 0.000, and *P*_0.01–0.1  versus  0.8_ = 0.000, Figure 3). But there were no differences in the three groups' range HM to 0.1. In the VA better than 0.1, the differences were significant in every group. The amplitudes of central point mfERG were increased gradually and significantly in each group (*P*_0.1–0.2  versus  0.5–0.8_ = 0.015, *P*_0.1–0.2  versus  0.8_ = 0.000; *P*_0.2–0.3  versus  0.5–0.8_ = 0.015, *P*_0.2–0.3  versus  0.8_ = 0.000; *P*_0.3–0.5  versus  0.8_ = 0.000; *P*_0.5–0.8  versus  0.8_ = 0.009; Figure 3).

### 3.4. Correlations between the PVEP, mfERG, and ERG Responses and the VA before Operation

**Figure 4:**
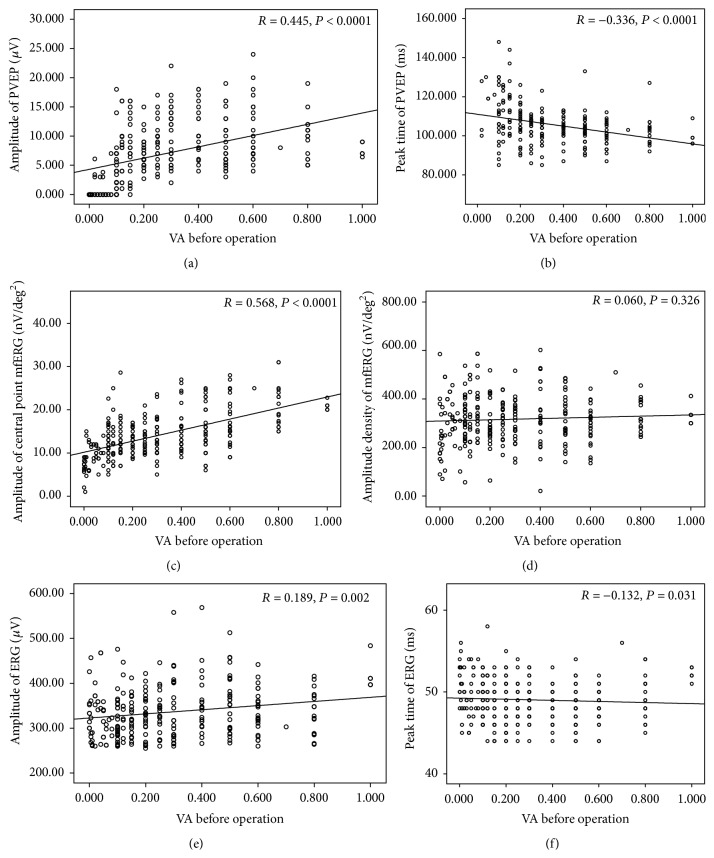
Correlations of the PVEP, mfERG, and ERG responses with the preoperative VA for patients with cataract. (a, b) Amplitude and peak time of PVEP, (c, d) amplitude of the central point, and amplitude density of mfERG. (e, f) Amplitude and peak time of b-wave dark-adapted 3.0 ERG. *P* is significant at 0.05 level.

The amplitude, central point mfERG, PVEP, and ERG responses correlated with preoperative VA but the amplitude density of mfERG did not (Figure 4). The relationship of the amplitude of central point mfERG with preoperative VA was 0.568, *P* < 0.0001, and the correlation of amplitude density of mfERG was 0.06, *P* = 0.326. The relationships between the amplitude and peak time of PVEP with preoperative VA were 0.445 and −0.336, respectively (*P*_Amplitude  of  PVEP : VA_ < 0.0001, *P*_Peak  time  of  PVEP : VA_ < 0.0001). The correlations of amplitude and peak time of the b-wave ERG with preoperative VA were 0.189 and −0.132 (*P*_Amplitude  of  b-wave : VA_ = 0.002, *P*_Peak  time  of  b-wave : VA_ = 0.031). To ensure the retinal function of the patients with cataract was good, the amplitude of the central point mfERG was the most relevant parameter when compared with the preoperative VA and PVEP and ERG responses.

### 3.5. Correlations between the PVEP, mfERG, and ERG Responses and the VA after Operation

**Figure 5:**
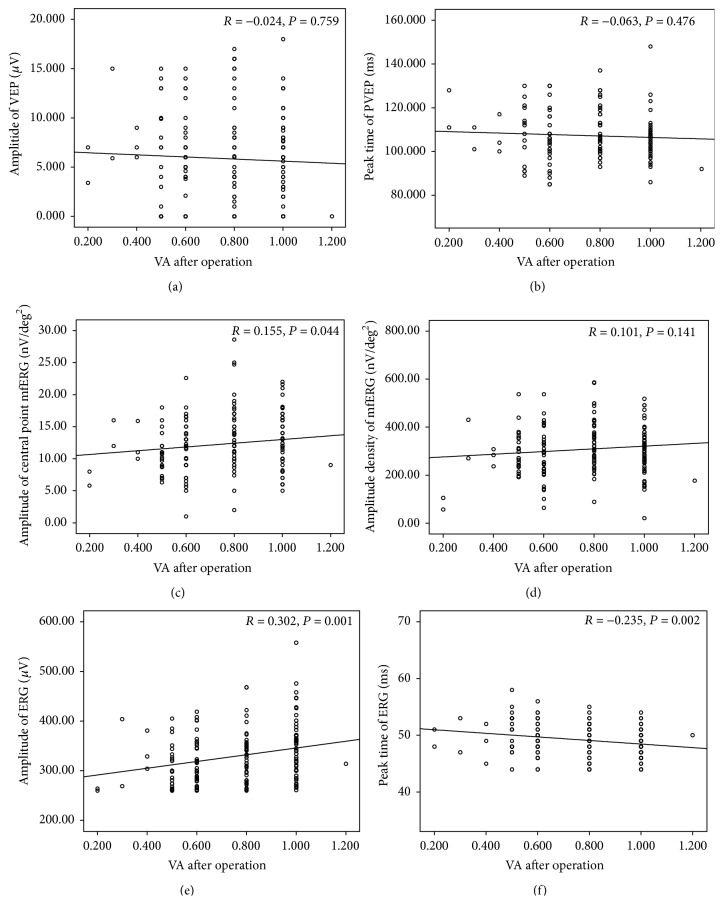
Correlations of the PVEP, mfERG, and ERG responses with the postoperative VA for patients with cataract. (a, b) Amplitude and peak time of PVEP, (c, d) amplitude of the central point, and amplitude density of mfERG. (e, f) Amplitude and peak time of b-wave dark-adapted 3.0 ERG. *P* is significant at 0.05 level.

The correlations of amplitude and peak time of PVEP with postoperative VA were −0.024 and −0.063, respectively (*P*_Amplitude  of  PVEP : post  VA_ = 0.759, *P*_Peak  time  of  PVEP : post  VA_ = 0.476). The correlation of the amplitude of the central point mfERG was 0.155, *P* = 0.044, and the correlation of the amplitude density of mfERG was 0.101, *P* = 0.141. The correlations of amplitude and peak time of the b-wave ERG with postoperative VA were 0.302 and −0.235 (*P*_Amplitude  of  b-wave : post  VA_ = 0.001, *P*_Peaktime  of  b-wave : post  VA_ = 0.002). The amplitude and peak time of ERG and amplitude of central point mfERG were statistically correlated with the postoperative VA (Figure 5). But the PVEP response was not significantly correlated with the postoperative VA.

### 3.6. Best Corrected VA of Cataract Patients before and after Surgery

There were 60 eyes whose postoperative VA was more than 0.5 and less than 0.8 and there were 104 eyes whose postoperative VA was more than 0.8. Only 7 eyes were less than VA of 0.5 after cataract surgery (Table 2). Over 95% (164/171 eyes) of the patients had a postoperative VA of more than 0.5. Only 4.1% (7/171 eyes) of patients suffering surgery were less than 0.5.

### 3.7. 95% CI of Amplitude of Central Point mfERG at Different Preoperative VA Levels

The amplitude of central point mfERG was more closely related to the preoperative VA. The 95% CI of amplitude of the central point mfERG was calculated in cataract patients grouped by preoperative VA levels (Table 3).

### 3.8. Postoperative VA

**Figure 6:**
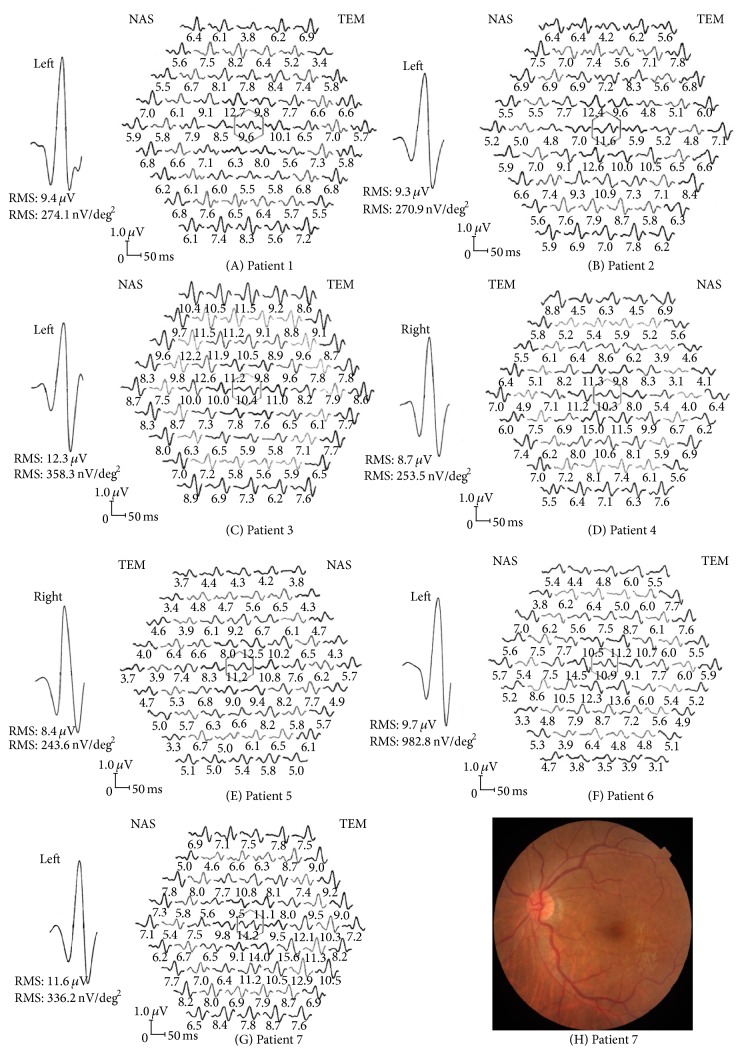
mfERG responses of cataract patients with low VA undergoing cataract surgery. (A-G) The amplitude of each point mfERG of 1 to 7 patients. (H) The fundus of patient 7 after cataract operation suffers from macular epiretinal membrane. NAS: nasal side; TEM: temporal side.

The postoperative VA of 7 eyes was less than 0.5. The postoperative VA in 2 patients was less than 0.3. Female patient number 1, aged 67 years, had a pre- and postoperative VA of 0.25, with a 95% CI of 0.3 > VA ≥ 0.2 of 12.46 to 14.18 nV/deg^2^. A 73-year-old female patient number 2 had a pre- and postoperative VA of 0.3 and a 95% CI of 0.5 > VA ≥ 0.3 of 13.30 to 16.22 nV/deg^2^. The amplitude of the central point mfERG was 9.6 nV/deg^2^ and 11.6 nV/deg^2^ for patients 1 and 2, respectively, which were less than the lower level value of the 95% CI (Figure 6).

The postoperative VA was 0.4 in 5 patients. A 77-year-old male patient number 3 had a preoperative VA of 0.25 and a postoperative VA of 0.4. The 95% CI of 0.3 > VA ≥ 0.2 was 12.46 to 14.18 nV/deg^2^; amplitude of central point mfERG was 10.4 nV/deg^2^. Patient 4, a female aged 67 years, had a VA before surgery of 0.2 and a VA after surgery of 0.4. The 95% CI of 0.3 > VA ≥ 0.2 was 12.46 to 14.18 nV/deg^2^; amplitude of central point mfERG was 10.3 nV/deg^2^. Patient 5, a male aged 78 years, had a VA before surgery of 0.2 and VA after surgery of 0.4. The 95% CI of 0.3 > VA ≥ 0.2 was 12.46 to 14.18 nV/deg^2^; amplitude of central point mfERG was 11.2 nV/deg^2^. Patient 6, male aged 77 years, had VA before surgery of 0.15 and VA after surgery of 0.4. The 95% CI of 0.2 > VA ≥ 0.1 was 11.86 to 14.19 nV/deg^2^; amplitude of central point mfERG was 10.9 nV/deg^2^. Patient 7, female aged 65 years, had VA before surgery of 0.2 and VA after surgery of 0.4. The 95% CI of 0.3 > VA ≥ 0.2 was 12.46 to 14.18 nV/deg^2^; amplitude of central point mfERG was 15.2 nV/deg^2^. The amplitudes of central point mfERG in the 6/7 patients were less than low line of 95% CI in different VA groups. The fundus examination of the first six patients showed the reflection of fovea cannot be seen. And the fundus of last patient showed macular epiretinal membrane (Table 4, Figure 6).

## 4. Discussion

The present study revealed that a well-defined amplitude of the central point mfERG was much more closely related to preoperative VAs than PVEP and ERG in agreement with a previous study [10]. They demonstrated that cataract seemed to reduce the central amplitude of the first positive peak (P1) and the second negative peak (N2) the most. The amplitude of peripheral mfERG measurements did not differ significantly pre- or postoperatively. Some other reports [11, 12] found that the amplitudes of N1 and P1 response from the central macula of patients with moderate cataract were notably reduced compared to that of patients with very mild cataract. But there was no significant difference in parafoveal responses in patients with cataract. We also found that there were no significant correlations between the amplitude densities of mfERG responses with the pre- or postoperative VA.

Compared with the amplitudes of PVEP, the amplitudes of central point mfERG were much more closely related to preoperative VA. PVEP was used to evaluate the postoperative visual function at an early period and it was suggested that it could be used to predict the outcome of cataract surgery before the removal of the opaque lens [5, 13, 14]. Recently, Fuest et al. used blue-yellow PVEP to test the ERG of patients before and after cataract surgery [15]. They demonstrated that the removal of the cataractous lens led to a significant reduction in the latency of the BY-VEP peaks. However, PVEP could misdiagnose macular degeneration [6]. Vernon Odom et al. [16] stated that patients with cataract and an acuity of 20/50 (0.4) or better have normal PVEPs in agreement with our data. We found that there was no significant difference between control and cataract groups in the PVEP amplitudes over the preoperative VA range 0.3 to 1.0 VA. And for the VA better than 0.2, the differences were not notable in cataract groups. Although the control group's mean age was 58.95 years while the cataract group's mean age was 65.73, there were not statistic differences in the response of retina function between the two groups (Table 1). In contrast to the correlation between mfERG and preoperative VA, the response amplitude of center point (2.17°) mfERG was significantly lower in the cataract group compared with the control group when the VA was less than 0.8. And the amplitudes of central point mfERG were increased gradually and significantly in almost each cataract group. The patients suffer from different degree simple cataract; they could get different level of amplitudes of central point mfERG. In view of the amplitude of the center mfERG was most correlated with preoperative VA in different degree cataract patients. Therefore, we calculated the 95% CI for the amplitude of center point mfERG for all of the cataract patient groups that were grouped by preoperative VA levels to evaluate the macular function.

The postoperative VA of seven eyes was less than 0.5. Under the 95% CI of center mfERG amplitudes, the values of 6/7 patients were below the range. The six patients suffered from macular edema (ME) after surgery. Only one subject was beyond the 95% CI range of 0.3 > AV ≥ 0.2. The fundus of the last patient showed macular epiretinal membrane. ME was the most frequent cause of limited visual recovery after cataract surgery. Mentes et al. showed that the incidence of clinical and angiographic cystoids macular edema (CME) after uncomplicated phacoemulsification was 9.1% [17]. Bélair et al. [18] showed that the incidence of CME was 4% of cataract patients without uveitis one month after surgery in agreement with our findings. In this study, the postoperative VA less than 0.5 was 4%. The incidence of macular edema was 3.5% in these patients after cataract operation with a low postoperative VA. And Chung et al. reported central mfERG response was suitable to evaluate CME due to branch retina vein occlusion [19]. Perhaps the amplitude of the center mfERG is an index sensitive to the patients who are prone to CME. That would be necessary for further considerations.

In our study almost 96% (164/171) of the patients that received cataract surgery resulted in an excellent postoperative VA. The ERG response was highly related to postoperative VA. We chose cataract patients without other ophthalmological and general diseases and chose patients whose amplitudes of ERG were more than 80% of normal database to ensure good retinal function. We found that the amplitude of the b-wave was closely correlated with postoperative VA for the scotopic 3.0 ERG response. However, some reports found that the ERG responses of subjects with cataract were lower compared with normal control subjects. Others have shown that there was no significant difference in amplitude of the b-wave between patients with cataract and control subjects [20, 21]. Knowing the function of the whole retina preoperatively is essential for postoperative VA. Over 95% of our patients had ideal outcome VAs greater than 0.5, higher than the range of 48%–95% (78% average) measured in other studies [22]. Some report that 90% of patients undergoing cataract extraction have postoperative acuities of 6/12 (20/40) or better [23]. The reason our percentage was higher is because we strictly chose to operate on patients depending on their preoperative ERGs. Thus, preoperative ERG is essential for determining whether the cataract surgery is necessary and will successfully improve VA of patients.

In conclusion, the amplitude of the central point mfERG was much more closely related and specific to different preoperative VA levels. The amplitude 95% CI of central mfERG could help to predict the outcome of cataract surgery at each level of preoperative VA. The function of whole retina is an essential precondition for cataract surgery. Visual electrophysiological tests indeed would be both time-consuming and costly before cataractous surgery. But the research has proved that ERG responses ensure a great function of whole retina and the mfERG assess the macular function. The diagnostic values of visual electrophysiological tests are needed and valuable before the cataract surgery in order to reduce unnecessary surgery and pain of patients. We also suggest that before cataract surgery each visual electrophysiology test room must determine their individual full field ERG and 95% CI of amplitude central mfERG to predict surgical outcome.

## Figures and Tables

**Table 1:** Normative amplitude values of b-wave in scotopic 3.0 ERG at different ages (40~83 y) in our lab.

Age (years)	Number (104)	Scotopic 3.0 ERG	
		Peak time of b-wave (ms)	Amplitude of b-wave (*μ*V)
40–49	42	48.6 ± 3.6	424 ± 123
50–59	32	48.0 ± 2.3	413 ± 78
60–69	24	47.7 ± 2.4	374 ± 64
≥70	6	49.2 ± 1.9	315 ± 45

**Table 2:** Best corrected standard logarithmic VA of cataract patients before and after surgery.

Group	Before operation	After operation	Operation
HM	8		
CF	6		
≥0.01, <0.1	30	2	N
≥0.1, <0.2	61	7	N
≥0.2, <0.3	47	11/2	N/Y
≥0.3, <0.5	51	24/5	N/Y
≥0.5, <0.8	52	40/60	N/Y
≥0.8	22	22/104	N/Y
Total	277	106/171	N/Y

Note: “N” denotes patient did not have cataract surgery; “Y” denotes patient had cataract surgery. HM: hand movements; CF: counting fingers.

**Table 3:** 95% CI of amplitude of central point mfERG at different preoperative standard logarithmic VA.

Visual acuity before operation	95% confidence interval for mean (nv/deg^2^)	
	2.5% lower bound	97.5% upper bound
HM	4.7153	8.2847
CF	3.5068	9.5265
≥0.01, <0.1	8.9112	10.9754
≥0.1, <0.2	11.8626	14.1931
≥0.2, <0.3	12.4575	14.1765
≥0.3, <0.5	13.3093	16.2240
≥0.5, <0.8	14.9387	18.0036
≥0.8	17.2275	23.2270
Control	15.25	22.3

Note: HM: hand movements; CF: counting fingers.

**Table 4:** VA after operation in cataract patients.

Patient #	Age (y)	Sex	Standard logarithmic VA before operation	Standard logarithmic VA after operation	Amplitude of central point mfERG (nv/deg^2^)	Eye of fundus
1	67	F	0.25	0.25	9.6↓	Macular edema
2	73	F	0.3	0.3	11.6↓	Macular edema
3	77	M	0.25	0.4	10.4↓	Macular edema
4	67	F	0.2	0.4	10.3↓	Macular edema
5	78	M	0.2	0.4	11.2↓	Macular edema
6	77	M	0.15	0.4	10.9↓	Macular edema
7	65	F	0.2	0.4	14.2	Macular epiretinal membrane

Note: VA: visual acuity; F: female; M: male.
